# Characteristics of HCV Co-Infection among HIV Infected Individuals from an Area with High Risk of Blood-Borne Infections in Central China

**DOI:** 10.1371/journal.pone.0094219

**Published:** 2014-04-07

**Authors:** Tiejun Zhang, Damien C. Tully, Sujuan Zhou, Na He

**Affiliations:** 1 Department of Epidemiology, School of Public Health, Fudan University, Shanghai, China and Key Laboratory of Public Health Safety (Fudan University), Ministry of Education, Shanghai, China; 2 Ragon Institute of MGH, MIT and Harvard University, Harvard Medical School, Boston, Massachusetts, United States of America; Johns Hopkins School of Public Health, United States of America

## Abstract

**Objective:**

Hepatitis C virus (HCV) and human immunodeficiency virus (HIV) co-infection has been proved to be a growing public health concern. The prevalence and genotypic pattern vary with geographic locations. Limited information is available to date with regard to HCV genotype and its clinical implications among those former commercial blood donor communities. The aims of this study were to genetically define the HCV genotype and associated clinical characteristics of HIV/HCV co-infected patients from a region with commercial blood donation history in central China.

**Methods:**

A cross sectional study, including 164 HIV infected subjects, was conducted in Shanxi province central China. Serum samples were collected and HCV antibody testing, AST and ALT testing were performed. Seropositive samples were further subjected to RT-PCR followed by direct sequence coupled with phylogenetic analysis of Core-E1 and NS5B regions performed in comparison with known reference genotypes.

**Findings:**

A total of 139 subjects were HCV antibody positive. Genotype could be determined for 88 isolates. Phylogenetic analysis revealed that the predominant circulating subtype was HCV 1b (65.9%), followed by HCV 2a (34.1%). The HCV viral load in the subjects infected with HIV1b was significantly higher than those infected with HCV 2a (P = 0.006). No significant difference for HCV RNA level was detected between ART status, CD4+ cell count level and HIV RNA level. Serum AST and ALT level were likely to increase with HCV RNA level, although no significance was observed. Those who had conducted commercial donation later than 1991 (OR 3.43, 95% CI: 1.12–10.48) and had a short duration of donation (OR 0.35, 95% CI: 0.13–0.96) were more likely to be infected with HCV 1b.

**Conclusion:**

These results suggest that HCV subtype 1b predominates in this population, and the impact of HIV status and ART on HCV disease progression is not significantly correlated.

## Introduction

Hepatitis C virus (HCV) was first identified in 1989 as the principal cause of post transfusion of non-A non-B hepatitis [1]. About 3% of the world population is infected with HCV, with a total of about 170 million carriers. Due to shared risk factors for transmission, co-infection with HIV and HCV is common [2]. Unregulated commercial blood collection among farmers occurred in central China, including Henan, Anhui and Shanxi provinces, during the 1990s resulting in the second major epidemic of HIV in the country. Previous studies have indicated that co-infection of HIV and HCV is very common in former blood donors [3], [4]. The first outbreak of HCV infection among plasma donors in China was reported in the last century [5], and since then the issue of HIV co-infection with HCV have been studied extensively, with most of the studies demonstrating high seroprevalence of HCV in this former commercial blood donors society [6], [7], [8], [9].

Understanding HIV/HCV co-infection and their clinical characteristics is of significant public health importance. Co-infection with HIV and HCV has been shown to increase HCV viral loads and may accelerate the natural course of chronic hepatitis C infection, increase the risk for the development of cirrhosis, hepatocellular carcinoma (HCC), and results in hastening progression to end-stage liver disease (ESLD) [10], [11], [12], [13], [14], [15], although whether co-infection HIV and HCV results in a faster progression to AIDS remains controversial [16], [17]. Unfortunately, such studies have rarely been done in China. Furthermore, combined intervention and treatment for HCV/HIV co-infection has not been taken into concerns and been rarely addressed.

HCV is a genetically diverse RNA virus with a single strand, positive sense genome, and is classified into 6 major genotypes with closely related isolates being grouped into subtypes [18], [19]. To date, genotypes 1, 2 and 3 have a worldwide distribution, while other genotypes have been found in more restricted regions only [20]. Genotyping is an important tool for epidemiologic studies, since HCV genotypes may vary according to epidemic history in different geographic regions with different genotypes manifesting substantial differences in clinical characteristics, such as response to treatment, which is of central importance in disease treatment and prevention. In China, the existing literature suggests that the prevalence of HCV genotype is highly heterogeneous with regard to infection sources and epidemic region. Over the years, despite the widespread evidence of co-infection with HIV and HCV, clinical characteristics of HCV/HIV co-infection in China has rarely specifically addressed, especially in terms of the association between HCV genotypes and clinical characteristics.

Therefore, a cross sectional study was designed and conducted to determine the prevalence of HCV genotypes within a sample of HIV infected patients from a rural Chinese community and to examine correlations between genotypes and the clinical implications, such as HCV RNA viral load distribution and serum enzyme levels, of these HCV/HIV co-infected patients. Our observations could bring new insight into the management of HIV/AIDS patients to reduce their morbidity and mortality.

## Material and Methods

### Ethics Statement

This study was approved by the Institutional Review Board of Fudan University, China. Written consent was obtained from all the adult participants before any procedures were performed. Interview data did not bear the names of respondents and all data were anonymized before analysis.

### Study sites and participants

The present study was conducted in a county in the southern part of Shanxi province in central China, which was once reported to have a high prevalence rate of HIV attributed to former commercial blood donors [7], [21], and harbors a large number of former commercial blood donors attributed to the establishment of an commercial blood donation center in 1995.

A total number of 164 HIV positive subjects who have been registered to the National HIV/AIDS Information System were randomly selected into the present study. Data for participants' socio-demographic characteristics and HIV transmission modes were extracted from the National HIV/AIDS Information System using a standard questionnaire form. Free antiretroviral treatment (ART) has been available for HIV/AIDS patients since 2003 in the study area.

### Serological testing

#### HCV serology

ELISA for Anti-HCV immunoglobulin G (IgG) antibody was tested to determine HCV infection status according to the manufacturer's protocol (Wantai Biomedical, Beijing, China). All the sera samples were assayed blindly in duplicate. Serum alanine aminotransferase (ALT) and aspartate aminotransferase (AST) were evaluated with commercial reagent kits (Shanghai Kehua Bioengineering Co. Ltd., China). Normal values were considered as: 10–40 IU/L for AST, and 5–40 IU/L for ALT.

### HCV RNA extraction

HCV-RNA extraction was carried out from 200 ml of serum using QIAamp1 Viral RNA Kit (QIAGEN, Valencia, CA), following the manufacturer's instructions. The synthesis of the complementary DNA (cDNA) was done immediately after RNA extraction and store −80°C. To avoid false-positive results, rigorous procedures proposed for nucleic acid amplification diagnostic techniques were followed.

### Complimentary DNA (cDNA) synthesis

Reverse transcriptase reaction was performed using the Moloney Murine Leukemia Virus Reverse Transcriptase (MMLV-RT) and random primers. The final volume of the reaction was 60 μl in the following concentrations: 50 mM Tris–HCl (pH 8.3), 75 mM KCl, 3 mM MgCl2, 10 mM DTT, 0.5 mM of each dNTP, 450 ng random primers, 30URNAse enzyme inhibitor (RNase OUTTM), and 300 U MMLV-RT. Samples were submitted to the following temperature cycles: 70°C for 10 min, 25°C for 15 min, 37°C for 60 min, and 95°C for 15 min in a thermocycler (Eppendorf Mastercycler 1, Eppendorf, Hamburg, Germany).

### Amplification & Sequencing of HCV NS5B, C/E1 region

#### Polymerase chain reaction (PCR)

Polymerase chain reactions (PCR) were done in two stages to increase sensitivity. HCV NS5B and C/E1 were amplified for genotyping and sequence analysis. Specific primers used for amplification and amplification cycles were as described [22]. The final PCR amplicons were separated by agarose gel electrophoresis, the correct bands were excised from agarose gel and extracted using the GFXTM PCR DNA and Gel Band purification kit (Amersham Bioscience UK Limited, Little Chalfont, United Kingdom). The second round PCR primers were used for the direct sequencing reactions using Bigdye® Terminator v3.1 Cycle Sequencing kit (Applied Biosystems, Foster City, CA) following the manufacturer's protocol. The resulting sequence data were checked by hand.

### Quantification of HCV RNA

serum HCV viral load was determined by using the one-step quantitative HCV RT-PCR kit (Shenzhen Piji Co. Ltd., China) with the standard-curve Taqman probe method. All tests were performed according to the manufacture protocol. (Piji Bio-Tech Company, Shenzhen., China)

### Phylogenetic Analysis

Sequence alignments were created using MUSCLE and manually edited [24]. Sequences were then classified into phylogenetic groups (i.e. genotypes and subtypes) using the REGA HCV subtyping tool (version 2.0, available at: www.bioafrica.net) [23]. In addition, maximum likelihood phylogenies were constructed using PhyML with reference strains obtained from the HCV sequence database (http://hcv.lanl.gov/content/hcv-db/index) provided by the Los Alamos National Laboratory with the following accession numbers: 1a.US.H77.NC_004102, 1a.US.HCV-H.M67463, 1a.US5003.EF407419, 1a.LTD6-2-XF224.AF511950, 1b.BR.03.EF032892, 1b.CNAY587016.AY587016, 1b.JPJT.D11355, 1c.IDHC-G9.D14853, 1c.INAY051292.AY051292, 1g.ES1804.AM910652, 2a.JPAY746460.AY746460, 2a.JPHC-J6.D00944, 2a.JPJFH-1.AB047639, 2b.JPHC-J8.D10988, 2b.JPUT971017.AB030907, 2b.MD2B-1.AF238486, 2c.BEBE1.D50409, 2i.VND54.DQ155561, 2j.VE.05.HM777359, 2j.VE.06.HM777358, 2k.MDVAT96.AB031663, 3a.DEHCVCENS1.X76918, 3a.CB.AF046866, 3a.NZL1.NC_009824, 3b.JPHCV-Tr.D49374, 3i.IN.02.FJ407092, 3k.IDJK049.D63821, 4a.EGED43.Y11604, 4a.USF7157.DQ418788, 4b.CAQC264.FJ462435, 4b.PTP026.FJ025854, 4b.PTP212.FJ025855, 4b.PTP245.FJ025856, 4c.CAQC381.FJ462436, 4d.CAQC382.FJ462437, 4d.ES24.DQ516083, 4d.US03-18.DQ418786, 4d.PS2.EU392172, 4f.FRIFBT84.EF589160, 4f.FRIFBT88.EF589161, 4f.CM_DAV9905.EU392169, 4f.CM_SP1578.EU392170, 4f.PS4.EU392174, 4f.PS6.EU392175, 4g.CAQC193.FJ462432, 4k.CAQC383.FJ462438, 4k.PB65185.EU392171, 4k.PS3.EU392173, 4l.CAQC274.FJ839870, 4m.CAQC249.FJ462433, 4n.CAQC97.FJ462441, 4o.CAQC93.FJ462440, 4p.CAQC139.FJ462431, 4q.CAQC262.FJ462434, 4r.CAQC384.FJ462439, 4t.CAQC155.FJ839869, 5a.GBEUH1480.NC_009826, 5a.ZASA13.AF064490, 6.CNGZ52557.DQ278892, 6a.HK6a35.DQ480513, 6b.Th580.NC_009827, 6c.THTh846.EF424629, 6d.VNVN235.D84263, 6e.CNGX004.DQ314805, 6f.THC-0044.DQ835760, 6g.HKHK6554.DQ314806, 6h.VNVN004.D84265, 6i.THC-0159.DQ835762, 6j.THC-0667.DQ835761, 6k.CNKM45.DQ278891, 6l.VND33.EU246933, 6m.THC-0185.DQ835765, 6n.CNKM42.AY878652, 6o.VND85.EU246934, 6p.CAQC216.EF424626, 6q.CAQC99.EF424625, 6r.CAQC245.EU408328, 6s.CAQC66.EU408329, 6t.VND49.EU246939, 6u.CNDH012.EU408330, 6v.CN.NK46.EU158186, 6w.TWHCV-6-D140.EU643834, 7a.CAQC69.EF108306.

### Statistical analysis

Original questionnaire data and laboratory results were entered and managed with EpiData 3.0 and then transferred to SAS v8.2 (SAS institute Inc., Cary, North Carolina, USA) for further analysis. Descriptive statistics and univariate analysis were performed to examine the correlates of HCV seropositivity. Multiple logistic regression analyses were conducted to identify potential factors associated with HCV subtype 1b infection compared to those with HCV subtype 2a infection. Crude odds ratio and odds ratio adjusted for gender and age group were generated to determine whether a variable was associated with HCV infection. All p-values ≤0.05 were considered to be statistically significant.

## Results

### Sociodemographic Characteristics of Study Participants

A total of 164 HIV positive subjects were finally recruited into the present study. Of them, 139 (84.7%) were HCV/HIV co-infected while 25 (15.3%) were HIV mono-infected (Table 1). Among the study subjects, those who participated in HCV/HIV co-infected group were more likely to be male (P = 0.019) and older (P = 0.013) (Table 1). No significant differences were reported between the co-infected and mono-infected groups in martial status, education, family income, smoking habits or consumption of alcohol. Eighty-six per cent of all participants acquired HIV from commercial blood donation while in the HIV mono-infected group transmission of HIV was more likely to occur from blood transfusion and sexual contact (24% vs. 3.6% for transfusion and 28% vs. 2.2% for sexual transmission). In addition approximately 78.7% of all study participants had received ART. The major demographic characteristics of all participants are summarized in Table 1.

**Table 1:** Socio demographic characteristics of HIV mono-infected and HIV/HCV co-infected subjects in the study.

Characteristics	HIV	HIV/HCV	Total
	N = 25	N = 139	N = 164
	No. (%)	No. (%)	No. (%)
**Gender** (P = 0.019)			
Male	11 (44.0)	95 (68.3)	106 (64.6)
Female	14 (56.0)	44 (31.7)	58 (35.4)
**Age group** (P = 0.013)			
≤20	2 (8.0)	1 (0.7)	3 (1.8)
21–49	20 (80.0)	98 (70.5)	118 (72.0)
≥50	3 (12.0)	40 (28.8)	43 (26.2)
**Marital status** (P = 0.147)			
Single	4 (16.0)	10 (7.2)	14 (8.5)
Ever married	21 (84.0)	129 (92.8)	150 (91.5)
**Education** (P = 0.460)			
Illiterate	2 (8.0)	13 (9.4)	15 (9.1)
Primary school	10 (40.0)	54 (36.8)	64 (39.0)
Middle school	10 (40.0)	66 (47.5)	76 (46.3)
Senior middle	3 (12.0)	5 (4.3)	9 (5.5)
**Family income** (P = 0.830)			
<1000	17 (68.0)	83 (59.7)	100 (61.0)
1001∼2000	3 (12.0)	20 (14.4)	23 (14.0)
2001∼3000	2 (8.0)	10 (7.2)	12 (7.3)
>3001	3 (12.0)	26 (18.7)	29 (17.7)
**Alcoholic Drink** (P = 0.329)			
Yes	3 (12.0)	9 (6.5)	12 (7.3)
No	22 (88.0)	130 (93.5)	152 (92.7)
**Smoking** (P = 0.152)			
Yes	8 (32.0)	66 (47.5)	74 (45.1)
No	17 (68.0)	73 (52.5)	90 (54.9)
**Routes of HIV transmission (P<0.001)**			
Commercial blood donation	13 (52.0)	126 (90.6)	139 (84.8)
Transfusion	4 (16.0)	6 (4.3)	10 (6.1)
Sexual transmitted	7 (28.0)	7 (5.0)	14 (6.1)
Mother to Child	1 (4.0)	0 (0.0)	1 (0.6)
**Antiretroviral therapy** (P = 0.052)			
Yes	16 (64.0)	113 (81.3)	129 (78.7)
No	9 (36.0)	26 (18.7)	35 (21.3)
**CD4+ counts** (P = 0.569)			
<200	7 (28.0)	47 (33.8)	54 (32.9)
≥200	18 (72.0)	92 (66.2)	110 (67.1)
**HIV viral load** (P = 0.946)			
<1000	7 (28.0)	38 (27.3)	45 (27.4)
≥1000	18 (72.0)	101 (72.7)	119 (72.6)

### Hepatitis C Virus Sequence Amplification and Classification

**Figure 1:**
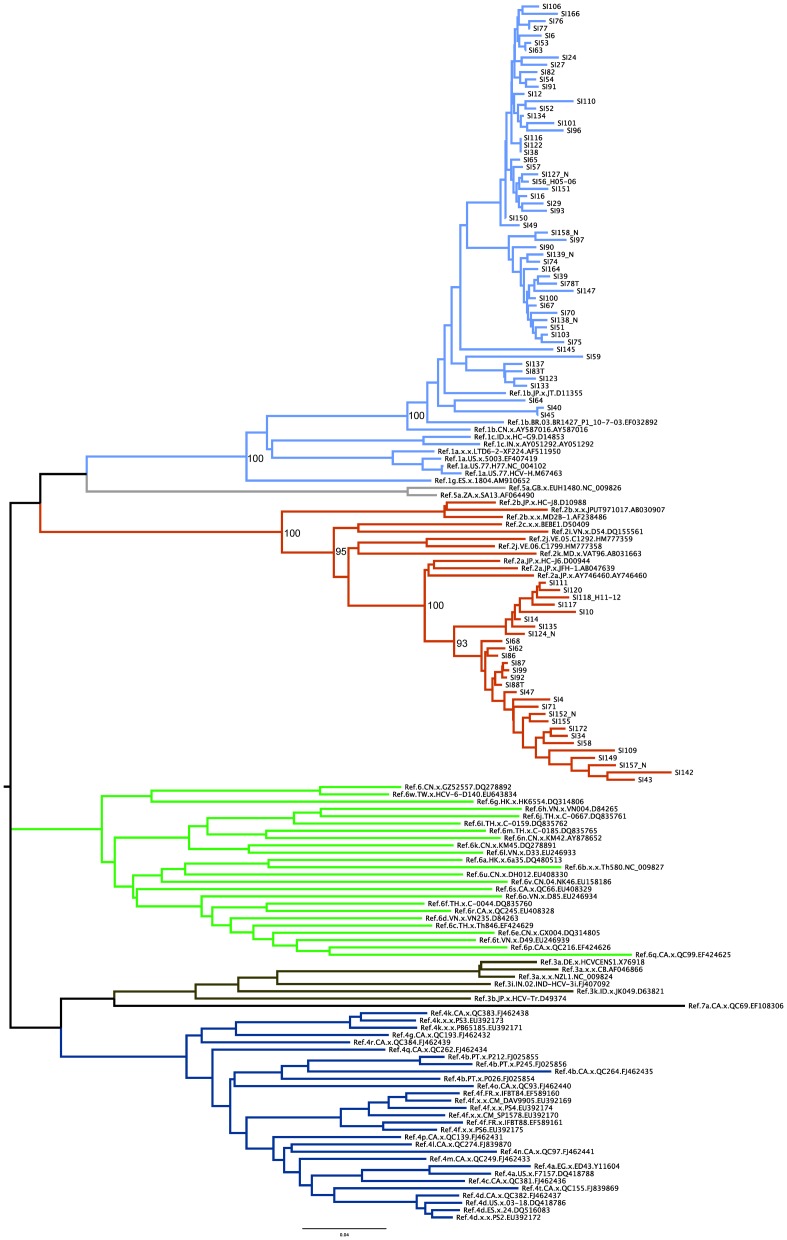
Maximum-Likelihood phylogeny estimated from NS5B sequences (H77 positions 8261–8639). Bootstrap support values are only shown for the major ancestral nodes of interest. Reference HCV sequences are indicated by genotype followed by their Genbank accession numbers. Similar phylogenetic patterns were observed for C/E1 (not shown).

Of the 139 HCV seropositive patients included in this study, 88 were also positive for HCV RNA. NS5B and C/E1 sequences were successfully amplified from 84 and 87 participants, respectively. HCV genotypes were determined using the REGA HCV Subtyping tool (version 2), which uses phylogenetic methods to identify the subtype against a panel of HCV subtype reference sequences. HCV genotyping was determined for the NS5B gene with 1b predominating in 56 individuals while 28 donors were found to be infected with genotype 2a (Figure 1). ML phylogenetic analysis also demonstrates significant clustering with high bootstrap supports (100%) for sequences belonging to the genotypes 1b and 2a clades respectively. REGA subtyping confirmed this observation for the C/E1 region with 57 patients infected with 1b and 30 infected with 2a. In addition, a similar phylogenetic pattern was observed for the C/E1 region with significant bootstrap support values (data not shown). No other genotypes were detected in this study. The genotyping results were consistent when analyzed with either NS5B or C/E1 region (measurement of agreement Kappa = 0.881, P<0.001).

### HCV viral load distribution

**Figure 2:**
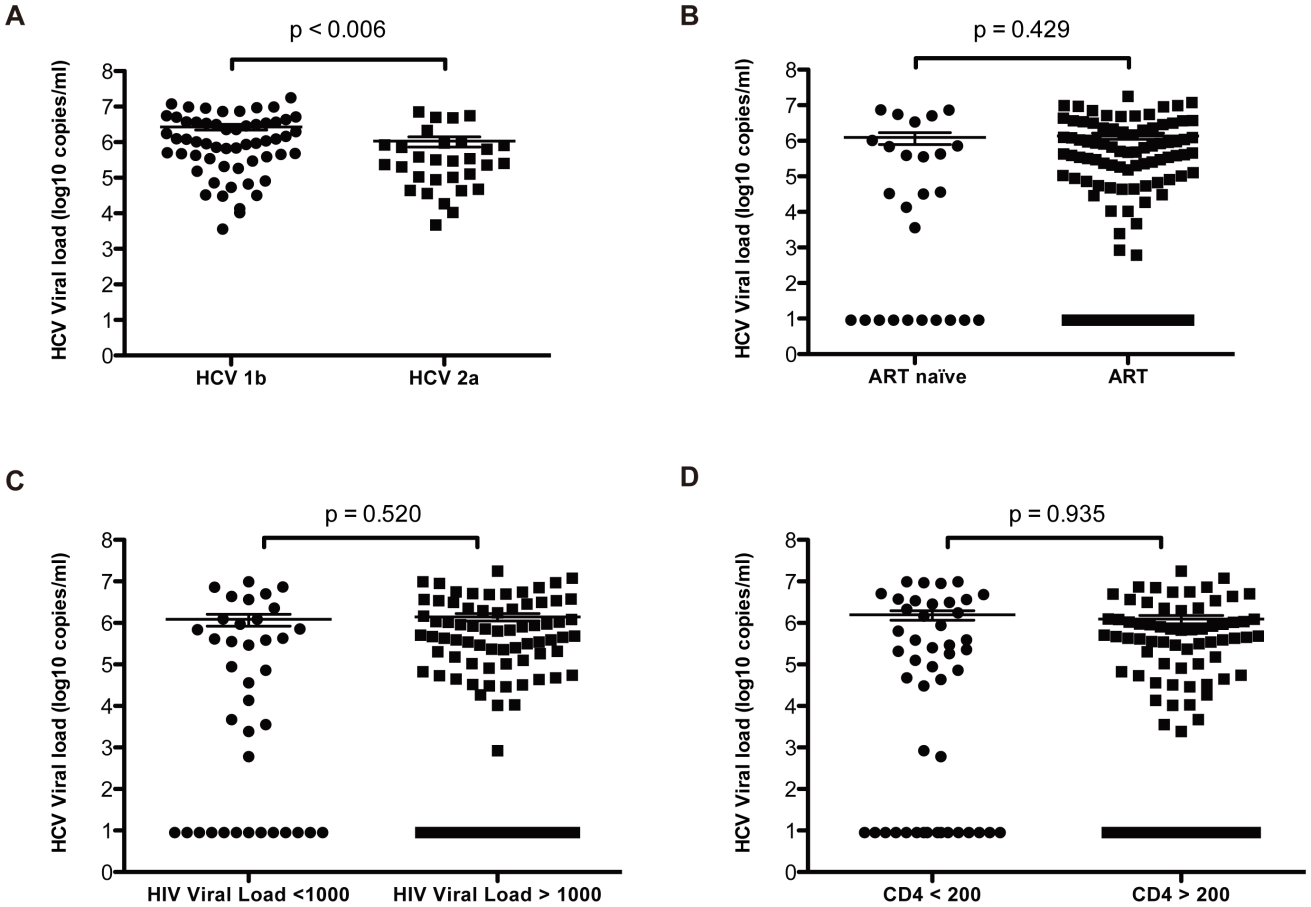
Box and Whisker Plots of HCV viral load distribution by related factors. (A) HCV viral load stratified by subtype 1b and 2a. (B) HCV viral load between ART naïve and ART treated subjects. (C) Distribution of HCV viral load by HIV viral load <1000 and >1000. (D) HCV viral load between CD4 counts <200 and CD4 counts >200.

Among those HCV RNA positive subjects, HCV viral loads were determined and compared. The overall median HCV viral load was 5.84log_10_copies/ml (interquartile range (IQR), 5.12–6.94 log_10_copies/ml). Furthermore, HCV viral load differences between genotypes, ART status, HIV viral load and CD4+count were explored. No significant differences were observed between the ART group (median 4.59 log_10_copies/ml; IQR, 0.95–5.89 log_10_copies/ml) and the ART naïve group (median 5.31 log_10_copies/ml; IQR, 0.95–6.06 log_10_copies/ml) (P = 0.429) (Figure 2). Similarly, HCV viral load was not significantly different between the HIV viral loads ≤1000 copies/ml group and HIV viral loads >1000 copies/ml group (P = 0.520) and low CD4 count group and high CD4 high counts group (P = 0.935). However, the HCV viral load of subtype 1b subjects (median 6.03 log_10_copies/ml; IQR, 5.50–6.56 log_10_copies/ml) was significantly higher than subtype 2a subjects (median 5.43; IQR, 4.87–5.93 log_10_copies/ml) (P = 0.006) (Figure 2).

### Relationship between HCV genotype and serum enzyme level

**Figure 3:**
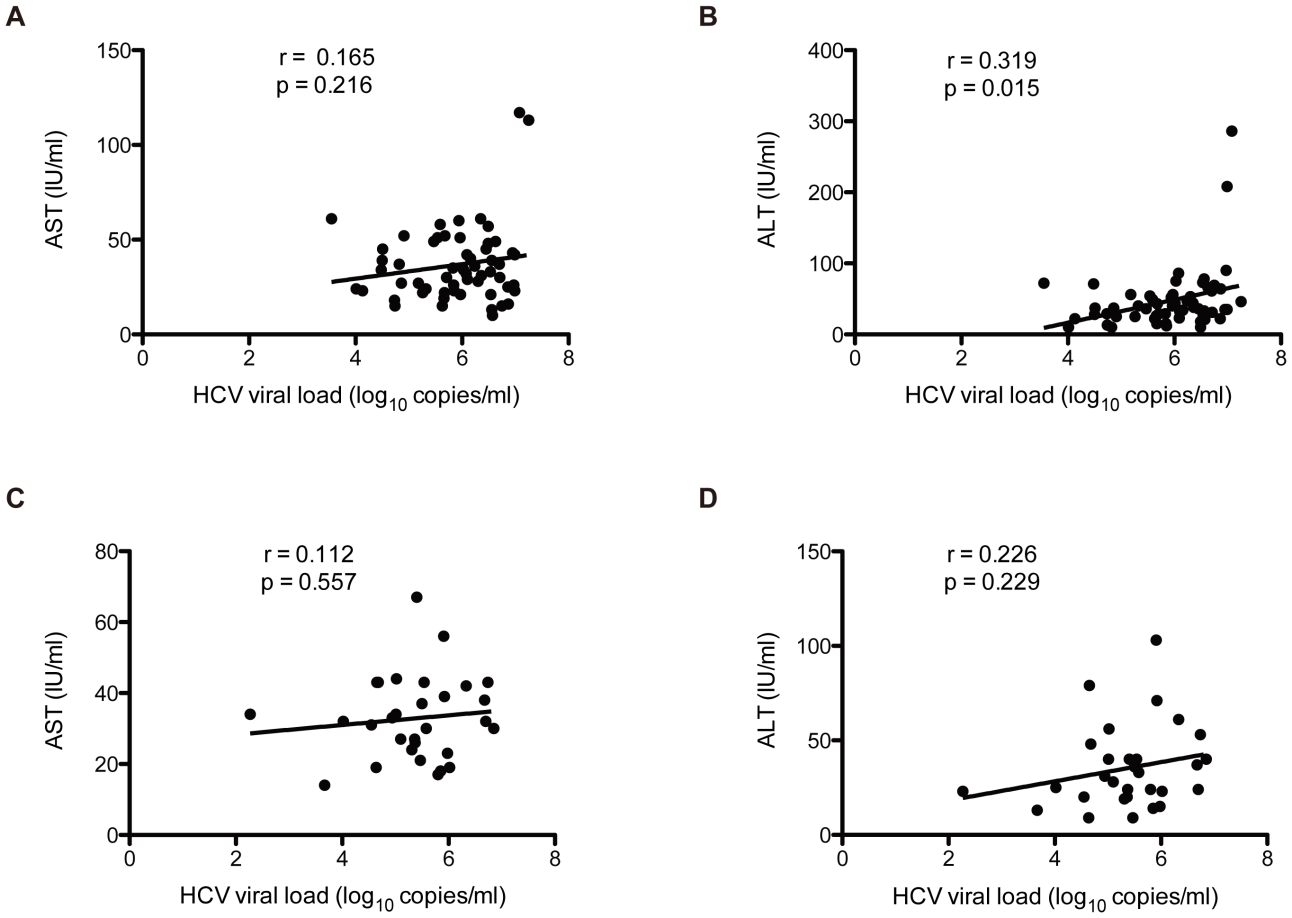
Correlation of serum enzyme levels and HCV viral load. (A) Association of AST and HCV viral load in subjects infected with subtype 1b. (B) Association of ALT and HCV viral load in subjects infected with subtype 1b. (C) Association of AST and HCV viral load in subjects infected with subtype 2a. (D) Association of ALT and HCV viral load in subjects infected with subtype 2a.

The serum AST and ALT levels were further analyzed for HCV genotypes 1b and 2a. The mean of AST and ALT were 36.72 UI/L and 47.21 UI/L for subtype 1b subjects, whereas the means of AST and ALT were 32.86 UI/L and 35.26 UI/L for subtype 2a subjects. As shown in Figure 3A and 3B, abnormal AST and ALT level (normal range 5–40 UI/L) were detected among 32.8% (19/58) and 41.4% (24/58) HCV subtype 1b infected subjects, with ALT were significantly increased with HCV viral load (P_AST_ = 0.216, P_ALT_ = 0.015). Similarly, serum AST and ALT level in the HCV subtype 2a infected subjects tended to be increased with HCV viral load though statistical significance was not observed (P_AST_ = 0.557 and P_ALT_ = 0.229), and the abnormal AST and ALT level frequency among subtype 2a subjects were 26.7% (8/30) and 23.3% (7/30), respectively.

### Potential risk factors associated with HCV subtype 1b infection

Analysis for potential risk factors was restricted to HCV subtype 1b and 2a cases. As shown in table 2, univariate analysis revealed that first donation time (P = 0.020), duration of commercial blood donation (P = 0.036) was significantly associated with subtype 1b infection. Multivariate Logistic analysis adjusted by age and gender indicated those who donated blood later than the year 1991 were more likely to be HCV subtype 1b infection (AOR = 3.43, 95% CI: 1.12–10.48), whereas if the duration of commercial blood donation was more than 3 years then the participants were less likely to be HCV 1b infection (AOR = 0.35, 95% CI: 0.13–0.96). No significant differences were found between subtype and route of transmission.

**Table 2:** Multivariate Logistic regression analysis of potential factors associated with HCV subtype 1b infection, compared to those with HCV subtype 2a infection (n = 88).

Characteristics/Risk factor	Subtype 2a (N = 30)	Subtype 1b (N = 58)		
	N (%)	N (%)	COR (95%CI)	AOR* (95%CI)
**First blood donation time** ^#^				
<1990	10 (34.5)	7 (12.5)	1.00	
≥1991	19 (65.5)	49 (87.5)	3.68 (1.22–11.08)	**3.43 (1.12**–**10.48)**
**Duration of commercial donation** ^#^				
<3	17 (58.6)	45 (80.4)	1.00	
≥3	12 (41.4)	11 (19.6)	0.35 (0.13–0.93)	**0.35 (0.13–0.96)**
**Routes of HIV transmission**				
Commercial blood donation	29 (96.7)	56 (96.6)	1.00	
Transfusion	1 (3.3)	2 (3.4)	1.03 (0.09–11.09)	0.92 (0.07–11.03)

# some subject have not donated.

*AOR adjusted for gender and age group.

## Discussion

During the early 1990s, commercial plasma and blood collection activities were once common in rural areas of central China and commercial donation for money seemed to be an easy way for those rural farmers to augment their income at that time [25]. However, due to the unhygienic process of pooling blood and the reinfusion of compatible red blood cells to permit more frequent donations prompted exposing the donors to a range of pathogens. The nature of such practices led to high HCV infections rates in blood and plasma donors with enhanced risk of HIV transmission in addition to other opportunistic infections [25], [26].

Several studies on HCV co-infection in former blood donors from other areas in China have shown similar results demonstrating that the HCV prevalence can be as high as 78.6% to 86.3% among HIV positive subjects [7], [8], [27]. Our results further confirm that dual HIV and HCV infection is relatively common. This is of public health importance, given that HCV co-infection may complicate, antiretroviral therapy (ART) and the use of the different regimens should be closely monitored in this former commercial blood donation region.

To elucidate the epidemiologic picture of circulating viral strains, HCV NS5B and C/E1, two reliable and most commonly used regions, were selected as targets to infer the genotype distribution in the present study [28]. The genotypes from two assays showed high consistency with no recombination detected. Overall, genotyping data showed that two main HCV genotypes, 1b and 2a, are circulating within those who are infected with HIV in central China. These results are in direct agreement with previous reports on HCV and HIV co-infection among commercial blood donor from neighboring provinces, such as Henan and Hubei in China [8], [29], [30]. Actually, subtype 1b and 2a are two of the most prevalent genotypes in China, with 1b prevailing in southern China while 2a is more common in northern China [29]. Interestingly, although a similar genotype pattern, consisting of subtypes 1b and 2a, was observed, the frequency of HCV subtypes differs across geographic regions. This altered distribution on genotype frequency may indeed corroborate recent reports that subtype 2a infections in China have been reduced [31].

The practice of risk behaviors is knowingly an important determinant of HCV transmission. Since the majority of study subjects had a history of commercial blood donation, HCV blood borne transmission should be of importance. In the current study, HCV 1b infection was associated with first donation time, while an inverse correlation has been observed from duration of commercial donation. It is speculated that subtype 1b has entered and become a predominate strain in this population after the year 1991. Conversely, those who had conducted commercial blood donation earlier (<1990) are more likely to be exposed to HCV subtype 2a.Moreover, those who and had a long duration of illegal blood donation often means donated earlier and are tend to be infected with subtype 2a.

To determine whether the HIV infection and ART affect nature course of chronic HCV infection, HCV viral load were compared between HIV RNA level, ART and CD4+ cell level. Currently, some discrepancy exists with previous data regarding HIV/HCV co-infection and the impact of ART on HCV progression [32], [33]. Data from the present study indicates that no significant difference was observed in HCV viral loads when the comparisons above were taken into account. Conversely, HCV viral loads were significantly higher in patients infected with subtype 1b than patients infected with subtype 2a (P = 0.006). There is discrepancy between our results and the studies by Liu et al. which indicated that patients infected with subtype 1b showed a lower HCV viral load compared with subtype 2a [30]. However, in general HCV 1b has been linked to severe chronic liver disease with results from this study supporting this fact that subtype 1b might be more aggressive and may be associated with high serum HCV levels.

Meanwhile, host responses of chronic HCV infection in those HIV positive subjects, in particular ALT and AST, have also been explored. Data from current study indicates that the majority of the HCV infected subjects' serum AST and ALT level are within normal range. Moreover, as previously been reported HCV viral load may not correlate with serum enzyme level in either subtype [34]. Furthermore, it is unlikely that the measurement of such enzymes at a single timepoint will be representive of the ALT/AST profile over time. Therefore, longitudinal data will better aid in supporting these conclusions.

In conclusion, the present study demonstrates that HCV/HIV co-infection is common in the former commercial blood donation community, with HCV 1b and 2a the two predominate subtypes. Although, HCV viral loads were higher in the subjects infected with subtype 1b than those who were infected with 2a, there is no correlation between HIV viral load, ART status, CD4+ cell counts, and HCV viral levels. Moreover, whether those specific subtypes could contribute to elevation of AST and ALT levels remains unclear. Prospective studies on HCV subtypes profile and clinical manifestation might be helpful in elucidating this understanding.
